# Response to “Assessing the outcomes of malaria intermittent preventive treatment during pregnancy on child growth trajectories”

**DOI:** 10.1016/j.ebiom.2024.105546

**Published:** 2025-01-07

**Authors:** Jade Benjamin-Chung, Yanwei Tong, Michelle E. Roh, Prasanna Jagannathan

**Affiliations:** a Department of Epidemiology and Population Health, Stanford University, Stanford, United States; b Chan Zuckerberg Biohub, San Francisco, United States; c Department of Statistics, Stanford University, Stanford, United States; d Institute for Global Health Sciences, University of California San Francisco, San Francisco, United States; e Department of Medicine, Stanford University, Stanford, United States

We appreciate the letter from Tang et al.^1^ regarding our manuscript.^2^ We agree that prenatal sexually transmitted infections (STIs) or reproductive tract infections (RTIs) are important causes of adverse pregnancy outcomes and could interact with malaria infections, with potential negative impacts on foetal and infant growth outcomes. It is also possible that sulfadoxine-pyrimethamine (SP), as a broad-spectrum antibiotic, can have an impact on STIs/RTIs and therefore could be a potential mediator as documented by a recent multi-country trial from Madanitsa et al.^3^ In that study, the authors reported that SP was associated with a lower risk of *Chlamydia trachomatis*.

Unfortunately, our study did not screen women for STIs or RTIs. Our dataset only contained information about STIs or RTIs using ICD codes, which could substantially underestimate true infection due to frequent asymptomatic infections. Understanding the mediating effect of STIs/RTIs and whether they interact with malaria infection in pregnancy to negatively impact foetal and infant health is an important area for future research. Given that we and Tang et al. posit that STIs/RTIs would be a mediator and/or effect modifier, accounting for them would only have changed our conclusions about evaluated mediators if there was strong, qualitative effect modification, which we consider to be extremely unlikely. Our results represent an average across any possible heterogeneous effects mediated by STIs/RTIs.

We agree that it is important to consider additional medications taken in conjunction with IPTp, and we have investigated this question using data from the trial in a prior study focused on non-malarial febrile infections.^4^ Regarding prescriptions for antibiotics for STIs/RTIs, no study participants received prescriptions through study clinics for azithromycin or for tetracycline, and only one received a prescription for doxycycline. 20% of women in the DP arm (n = 74) and 19% of women in the SP arm (n = 80) took metronidazole (p = 0.59); given that the intervention did not influence metronidazole use, it is not possible that it could have been a mediator of intervention effects.

With regard to anaemia, we agree with Tang et al. that it could be an important mediator; however, our original analysis found no evidence of mediation by anaemia in pregnancy. We also agree that prenatal iron supplementation could be a confounder of the relationship between maternal anaemia, placental malaria, and child growth outcomes. If such confounding was present, it could have influenced our mediation results since our models did not adjust for it.

We performed additional analyses to investigate the association between the number of ferrous sulfate tablets mothers consumed during the study and these variables using generalised linear models with a Poisson family for binary outcomes and a Gaussian family for continuous outcomes.

We did not observe an effect of DP vs. SP on iron supplementation; the mean number of ferrous sulfate tablets taken in pregnancy was 6.5 in the SP arm and 6.6 in the DP arm (p = 1.00). We found no associations between ferrous sulfate consumption in pregnancy and placental malaria (prevalence ratio = 0.97, 95% CI 0.88, 1.07). The number of ferrous sulfate prescriptions in pregnancy was positively associated with ever having anaemia during study follow-up (prevalence ratio = 1.26; 95% CI 1.18, 1.34). We found no association with weight-for-length Z in the first 6 months of life (Table 1); the number of ferrous sulfate doses consumed in pregnancy was associated with length-for-age Z from birth to 3 months, with a stronger association at birth.

**Table 1:** Mean difference (95% CI) in child growth Z-scores for each additional tablet of ferrous sulfate taken by their mother in pregnancy.

	Length-for-age Z	Weight-for-length Z
Birth	0.182 (0.112, 0.253)	0.000 (−0.088, 0.089)
1 day to 3 months	0.005 (0.001, 0.009)	0.001 (−0.003, 0.005)
3–6 months	0.000 (−0.004, 0.004)	0.000 (−0.004, 0.004)

To investigate the influence of iron supplementation on the mediated effects on length-for-age Z, we repeated mediation analyses including the number of ferrous sulfate tablets mothers consumed during the study as a covariate. Results were nearly the same from birth through age 3 months (Fig. 1). Evidence for mediation of IPTp by birthweight was slightly stronger and statistically significant from 3 to 9 months.

**Fig. 1:**
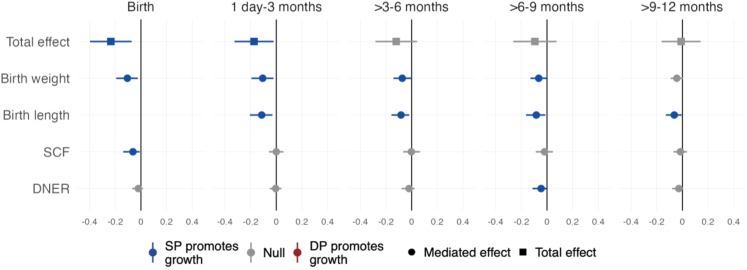
**Total and mediated effects of IPTp-DP vs. IPTp-SP on length-for-age Z-score**. The total effects compare mean Z-scores between IPTp-DP and IPTp-SP using unadjusted models. The figure includes mediators for which there was a mediated effect with a 95% CI that did not cross the null at any age. All mediated effects of IPTp-DP vs. IPTp-SP on Z-scores were adjusted by infant sex, maternal age, maternal baseline parasitaemia, gestational age at enrolment, maternal education, household wealth, and gravidity. The mediated effects at birth and 1 day–3 months were also adjusted by prenatal iron supplementation. The mediated effects through birth length, birth weight, LBW, and preterm birth were adjusted by anaemia. The reference group was SP. Includes all gravidae. For non-Olink mediators, the mediated effects include data from N = 608 children at birth, N ∈ [591, 593] from 1 day to 3 months, N ∈ [573, 575] from >3 to 6 months, N ∈ [557, 559] from >6 to 9 months, and N ∈ [547, 548] from >9 to 12 months. For Olink mediators, the mediated effects includes data from N = 255 children at birth, N = 251 from 1 day to 3 months, N = 255 from >3 to 6 months, N = 255 from >6 to 9 months, and N = 255 >9–12 months. The sample sizes for total effects is 622. SCF (KITLG) = Stem cell factor (Kit ligand).

We conclude that while iron supplementation is likely an important part of the causal process, in our particular study, accounting for it did not have a strong influence on our findings.

The total effects compare mean Z-scores between IPTp-DP and IPTp-SP using unadjusted models. The figure includes mediators for which there was a mediated effect with a 95% CI that did not cross the null at any age. All mediated effects of IPTp-DP vs. IPTp-SP on Z-scores were adjusted by infant sex, maternal age, maternal baseline parasitaemia, gestational age at enrolment, maternal education, household wealth, and gravidity. The mediated effects at birth and 1 day-3 months were also adjusted by prenatal iron supplementation. The mediated effects through birth length, birth weight, LBW, and preterm birth were adjusted by anaemia. The reference group was SP. Includes all gravidae. For non-Olink mediators, the mediated effects include data from N = 608 children at birth, N ∈ [591, 593] from 1 day to 3 months, N ∈ [573, 575] from >3 to 6 months, N ∈ [557, 559] from >6 to 9 months, and N ∈ [547, 548] from >9 to 12 months. For Olink mediators, the mediated effects includes data from N = 255 children at birth, N = 251 from 1 day to 3 months, N = 255 from >3 to 6 months, N = 255 from >6 to 9 months, and N = 255 > 9–12 months. The sample sizes for total effects is 622. SCF (KITLG) = Stem cell factor (Kit ligand).

## Contributors

YT analysed the data. JBC, YT, MER, and PJ wrote the first draft of the manuscript and reviewed and approved it. YT and JBC accessed and verified the underlying data.

## Declaration of interests

The authors declare no potential conflicts of interest.
